# LOTUS Software to Process Wearable EmbracePlus Data

**DOI:** 10.3390/s24237462

**Published:** 2024-11-22

**Authors:** Jack S. Fogarty

**Affiliations:** Science of Learning in Education Centre, National Institute of Education, Nanyang Technological University, Singapore 637616, Singapore; jack.fogarty@nie.edu.sg

**Keywords:** Empatica, EmbracePlus, wearables, physiological signal processing, software

## Abstract

The Empatica EmbracePlus is a recent innovation in medical-grade wristband wearable sensors that enable unobtrusive continuous measurement of pulse rate, electrodermal activity, skin temperature, and various accelerometry-based actigraphy measures using a minimalistic smartwatch design. The advantage of this lightweight wearable is the potential for holistic longitudinal recording and monitoring of physiological processes that index a suite of autonomic functions, as well as to provide ecologically valid insights into human behaviour, health, physical activity, and psychophysiological processes. Given the longitudinal nature of wearable recordings, EmbracePlus data collection is managed by storing raw timeseries in short ‘chunks’ in avro file format organised by universal standard time. This is memory-efficient but requires programming expertise to compile the raw data into continuous file formats that can be processed using standard techniques. Currently, there are no accessible tools available to compile and analyse raw EmbracePlus data over user-defined time periods. To address that, we introduce the LOTUS toolkit, an open-source graphical user interface that allows users to reconstitute and process EmbracePlus datasets over select time intervals. LOTUS is available on GitHub, and currently allows users to compile raw EmbracePlus data into unified timeseries stored in more familiar Excel or Matlab file formats to facilitate signal processing and analysis. Future work will expand the toolkit to process Empatica E4 and other wearable signal data, while also integrating more sophisticated functions for feature extraction and analysis.

## 1. Introduction

LOTUS is an open-source Matlab toolkit for longitudinal timeseries unification and signal processing, featuring a graphical user interface (GUI) to read, aggregate, and process wearable signal data from the Empatica EmbracePlus (https://github.com/jack-fogarty/LOTUS, accessed on 6 October 2024). The EmbracePlus is an advanced research-grade smartwatch that records continuous physiological and actigraphy data using a range of wrist-based sensors, including measures of cardiovascular activity via an optical photoplethysmogram (PPG), electrodermal activity (EDA), peripheral skin temperature, and three-axis accelerometry. This technology holds significant value by allowing the integration of real-time digital health monitoring tools into daily life. However, raw data from the device can be difficult to access and manage without purpose-built software. LOTUS aims to address this by offering researchers an easy-to-use toolkit to selectively output and interact with raw EmbracePlus data and apply standard signal preprocessing techniques.

EmbracePlus was released by Empatica in 2022 to replace their popular but now discontinued E4 watch. This new model records data similar to the E4, but with changes to the design, user interface, and output data management. In terms of data management, participants’ data may be downloaded either as a CSV with biomarkers aggregated over each minute of the recording, or as raw signal data with higher sampling frequencies. Notably, raw signal data are only available in avro file format, with each file storing up to 30 min of signal data organised by universal standard time. A challenge for users, then, is that avro files are not easily read or reformatted for standard use without some programming experience. The variable and fragmented storage of timeseries data in ‘chunks’ also calls for the data to be reconstituted before it may be processed. This can be arduous, particularly if users wish to focus on specific periods in dense longitudinal datasets. To our knowledge, there is no software to selectively reconstitute and process EmbracePlus data. The subsequent paragraphs provide a brief review of the literature using EmbracePlus, prior to introducing LOTUS to address this gap.

A search for ‘EmbracePlus’ on Google Scholar, Scopus, and PubMed identified 19 peer-reviewed studies using the EmbracePlus, along with 12 study protocols and 6 dissertations published between 2022 and 2024 (search date: 29 August 2024; records, 120; results, [1,2,3,4,5,6,7,8,9,10,11,12,13,14,15,16,17,18,19,20,21,22,23,24,25,26,27,28,29,30,31,32,33,34,35,36,37]). The diversity of these studies illustrates the wide-ranging applications for wearable research, with EmbracePlus used to monitor clinical health markers, fatigue, stress, emotion, and arousal in lab spaces, simulated work environments, or real-world settings. Most studies focused on adults (n = 21) [1,2,3,4,5,6,7,8,9,10,12,13,15,16,18,32,33,34,35,36,37], with few in older adult or clinical groups (n = 6) [3,7,8,11,17,18], and few with children (n = 3) [11,14,19]. Cardiovascular measures were most commonly used for analysis (n = 28) [1,2,3,4,5,7,8,9,10,11,12,13,14,15,17,21,22,23,25,27,28,29,32,33,34,35], followed by electrodermal activity (n = 25) [1,2,3,4,5,8,9,10,11,13,14,16,17,19,20,21,22,24,25,27,28,29,32,33,34], temperature (n = 20) [1,3,4,5,7,9,10,13,14,16,17,18,19,20,22,24,25,27,28,29], and accelerometry measures (n = 12) [8,15,16,20,22,23,24,25,27,28,29,37]. Fewer studies analysed actigraphy, respiration, or other biomarkers [6,7,8,20,23,24,25,27,28,29,35,36].

The previous literature shows promising outcomes for using EmbracePlus to measure stress or emotional arousal in realistic settings [2,3,10,12,14,19] or to enhance model accuracy for human activity recognition using statistical or machine learning methods [4,5,8,13,16]. Further utility of the EmbracePlus was also shown in studies demonstrating feasibility for health monitoring [6,7,17]. Notably, however, there appears to be no clear standard for data processing across studies.

The present review found there to be no complete software developed for EmbracePlus data processing. Indeed, most studies did not report the tools and methods used for processing EmbracePlus data. Several studies indicated the use of Matlab, R, and Python for data analysis, but even so, it is often still unclear how the raw or aggregate data were first compiled or processed to enable quantification [2,3,12,15,19]. Empatica provides a Python script to convert avro files to CSV. However, this is a direct conversion without any restructuring or analysis. Previous studies either only used aggregate data or are assumed to have used this Python script, along with custom code, to analyse signals from the EmbracePlus.

Three relevant toolkits were identified when searching GitHub repositories for ‘EmbracePlus’, with each toolkit being in development and having only limited capabilities with the raw avro data (Table 1). Wearables shows promise for providing a comprehensive processing suite, but this was developed for the E4 [38] and currently only reads EmbracePlus data. EmpaticaR can read avro files into nested R data frames but does not reconstitute the timeseries, while the EmbracePlus-Visualiser allows users to upload avro files to be compiled and downloaded as a CSV, but this web-based app handles limited amounts of avro data and does not enable further processing.

Software to process EmbracePlus data is needed to facilitate and encourage some standardisation of data processing in this growing field of wearable research. LOTUS was designed to address this gap and to provide researchers with a flexible easy-to-use tool to process Empatica data, with the capacity to build in additional functions and leverage Matlab’s powerful signal processing capabilities to drive wearable data processing and analyses. LOTUS is described in detail in the following section, with an emphasis on its basic layout and operation.

## 2. Software Description

LOTUS is an open-source Matlab toolkit developed in Matlab 2023b (The MathWorks). The toolkit features two GUI components: (i) the LOTUS reader and (ii) the LOTUS analyser. Each component is compatible with Windows and Mac OS, and compiled versions are available so that users without Matlab can freely use the toolkit. LOTUS is managed via GitHub for secure versioning, troubleshooting, and contributions for optimisation and development. It is currently capable of handling raw EmbracePlus EDA, BVP, systolic peak, temperature, and accelerometry data, with extensions for additional data and wearable input being developed for future releases.

### 2.1. LOTUS Reader

**Figure 1:**
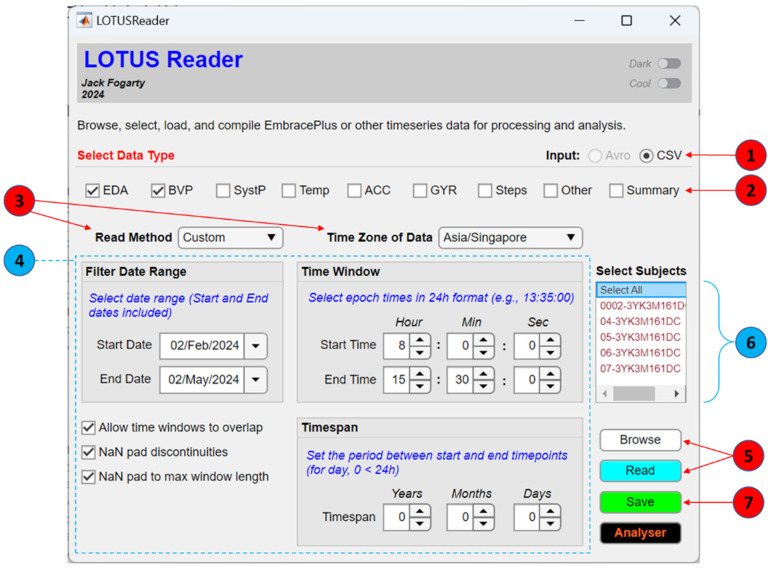
The GUI for the LOTUS reader. Workflow: (1) select input format, (2) select desired modalities, (3) choose the read method and time zone, (4) set any required time constraints, (5) browse the participant_data folder and then click read, (6) select the participants, and (7) save.

The LOTUS reader is designed to selectively read and compile raw EmbracePlus data and output the reconstituted signals in mat files that can be processed in the LOTUS analyser or other programs. This reader is flexible when handling Empatica EmbracePlus data, allowing users to specify parameters like the input file format (i.e., avro or CSV), for cases where the datasets have or have not already undergone a conversion. Figure 1 shows the GUI and basic workflow for the LOTUS reader, involving a single-panel interface allowing users to specify the read settings.

#### 2.1.1. Using LOTUS Reader

**Figure 2:**
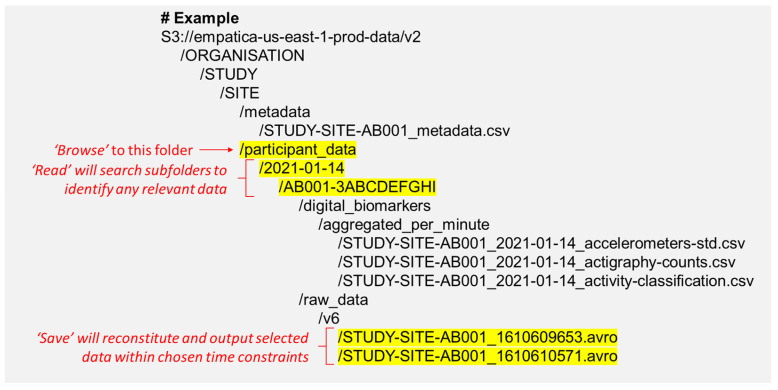
Example of a folder and file structure from the EmbracePlus manual [39], annotated to show where to browse for LOTUS input. After browsing to the participant_data folder, clicking the read button will identify the relevant data and update the subject list.

This section describes the LOTUS reader’s usage and settings. A pictorial guide to using this software is also available in the GitHub repository. To utilise the reader, first open the app by calling the *LOTUS* function in Matlab or loading the execution file. Once open, users can set up their input and read method, browse to their participant_data folder, click read, select the required subjects, and then save. LOTUS uses an algorithm to automatically identify and piece together relevant input data, following the conventional folder and file structures that would be downloaded from the Empatica Care Portal (Figure 2). That is, once the user browses to their ‘participant_data’ folder, clicking read will prompt LOTUS to identify the subfolders containing participants’ data according to the subfolders’ names, organised first by date (i.e., YYYY-MM-DD), then by subject–device identifiers. Folders that do not fit this structure are ignored; hence, it is recommended to maintain (or construct) this folder hierarchy when storing raw data.

##### Workflow and Settings

The workflow for reading and compiling data is summarised in Figure 1. First, users should specify whether they wish to read raw data from avro or CSV files (Figure 1, Step 1). The users must then select the type of data they wish to output by checking the boxes next to the relevant data, such as EDA, BVP, systolic peaks (SystP), temperature (Temp), or accelerometry (ACC) (Figure 1, Step 2). The read method and time zone for the output should then be set (Figure 1, Step 3). Specifying the time zone enables the raw data to be converted from universal standard time to a more interpretable format. It is recommended to use the time zone in which the data were originally recorded; however, this is at the discretion of the user.

Regarding the read method, users may select either a default or custom method. The default method reads and reconstructs individual subject data for each date in the participant_data folder, reconstituting all the available data across the entire day for each subject. In contrast, the custom method allows users to specify a time window to selectively output data within a given period relative to the chosen time zone (e.g., 11 a.m. to 1:30 p.m.). A timespan can also be set to indicate whether the selected time window should span more than 24 h (e.g., 11 a.m. to 1:30 p.m. the next day). If choosing the custom read method, users should proceed to specify their time window and (if necessary) their time span in the relevant panels (Figure 1, Step 4).

Additional read settings include an option to filter the date range for the default and custom read methods. This will restrict input/output to data that are available within the selected date range, which can be advantageous when navigating longitudinal datasets or needing to isolate specific data. Users can also choose to allow time windows to overlap, NaN pad discontinuities, or NaN pad out to max window length. Overlap applies when a set time span is over 24 h; for example, outputting data from 9:30 a.m. to 10:30 a.m. the next day could result in 1 h of overlap for recordings over consecutive days. If overlap is not allowed, then LOTUS will skip forward and continue with the nearest start time that does not overlap with a previously identified time window. NaN pad discontinuities and max window length pad out any missing data in the chosen time period with empty cells; these settings can help to ensure continuity and that participants’ timeseries are of relatively equal length, respectively.

**Figure 3:**
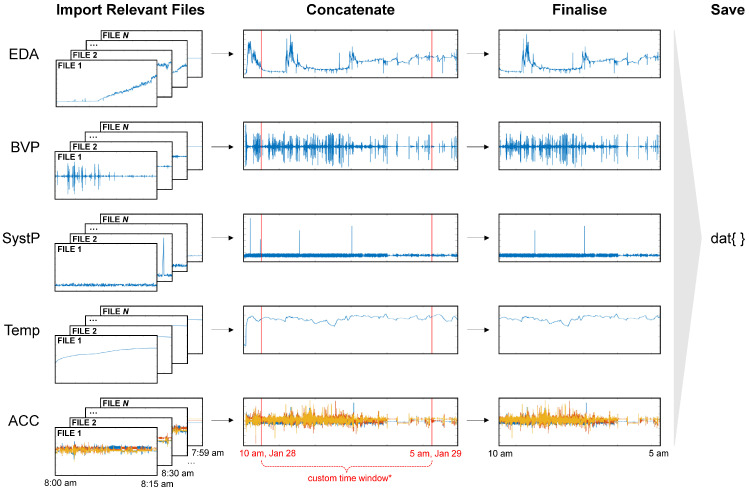
Data reconstitution in the LOTUS reader. EmbracePlus signal data including EDA, BVP, systolic peaks (SystP), temperature (Temp), and accelerometry (ACC) were recorded for 24 h, starting at 8 a.m. (stored in 96 avro files). Relevant avro files are then imported via LOTUS, concatenated, and then trimmed to a ‘custom’ time window set by the user (* setting only relevant for the custom read method; e.g., 10 a.m. 28 January to 5 a.m. 29 January). The final continuous data are then output as a mat file (i.e., dat).

After the user has entered the desired settings, they may then click ‘browse’, navigate to their participant_data folder, and then click ‘read’ to update the cache of subject data (Figure 1, Step 5). The data that are available with the chosen settings should be visible in the subject list, allowing users to then select all or specific subjects for output (Figure 1, Step 6). Following subject selection, users should click ‘save’ to output raw reconstituted data (Figure 1, Step 7). As illustrated in Figure 3, signal reconstitution simply involves the concatenation of the continuous data identified between the start and end of the chosen time period, according to the read method and settings.

#### 2.1.2. Output of the LOTUS Reader

The LOTUS reader allows for batch import and export of EmbracePlus data and will provide a single mat file for each individual recording that fits the chosen time constraints (i.e., read settings). Each mat file contains two structures: (i) cfg and (ii) dat. The cfg or ‘configuration’ structure contains information on the selected file parameters for the user’s reference and use in LOTUS analyser. The dat or ‘data’ structure contains the reconstituted signal data, as well as any event tags (i.e., markers) and a summary of the total duration, points, and percent of missing data for each requested signal.

**Figure 4:**
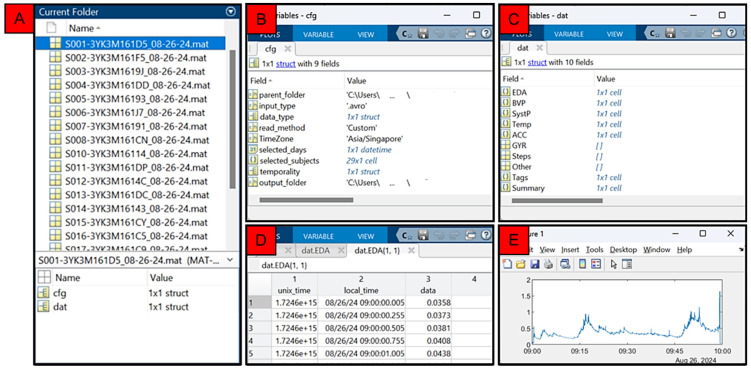
Typical output from the LOTUS reader. Panel (**A**): Mat files storing output from LOTUS after reconstituting avro data for a group of subjects (e.g., S001, S002, etc). Panel (**B**): example contents of a cfg file stored in one of the mat files shown in Panel (**A**). Panel (**C**): example contents of the dat file structure stored in a mat file shown in Panel (**A**). Panel (**D**,**E**): example of raw EDA timeseries stored in the output.

Figure 4 shows an example of the output generated using the custom read method to read EDA, BVP, systolic peak, temperature, and accelerometry data between 9 and 10 a.m. for several participants (i.e., S001, S002, etc.). Mat files are generated for each unique recording and labelled with the subject–device identifier and start date for the extracted timeseries, and contain a cfg and dat structure (Figure 4, Panel A). The cfg structure contains information regarding the parent folder for the input, the input and output type selected, the read method, the time zone, and other metadata (Figure 4, Panel B). The dat structure contains cells housing the raw continuous data, along with a table detailing the event tags, and a data summary (Figure 4, Panel C). Timeseries data in each cell are stored as a table with columns for universal standard time, local time (according to the selected time zone), and the raw data; an example of EDA data for one subject is shown in Figure 4, Panels D and E. Data from the other modalities (e.g., BVP, SystP, Temp, ACC) are output across the same timeframe, albeit at their given sample rate (e.g., Figure 3).

### 2.2. LOTUS Analyser

**Figure 5:**
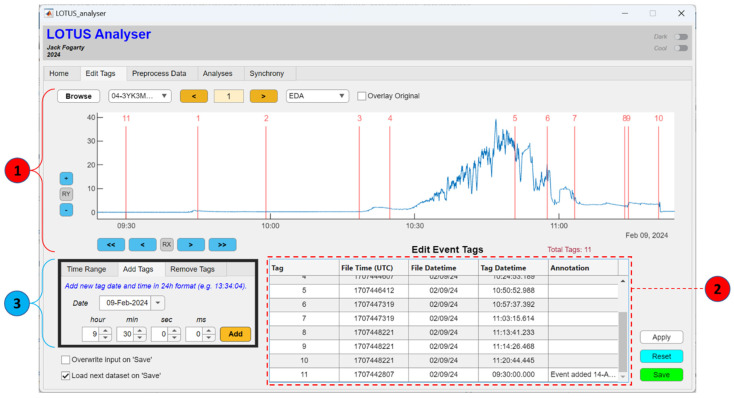
The LOTUS analyser, with the Edit Tags tab displaying reconstituted EDA data from a dat file output by LOTUS reader. The central plot displays the EDA timeseries (blue line), with red vertical lines showing the tags, consistent with those listed in the table and the input dat file. (1) Common tab controls to browse, load, and visualise data. (2) Event tag table. (3) Panel controls to add and remove event tags.

The LOTUS analyser aims to provide a comprehensive set of tools for researchers to interactively process and analyse wearable data. The GUI for the analyser is shown in Figure 4 and Figure 5, featuring several tabs with functions that allow users to load, visualise, and navigate raw data, edit tags, conduct standard preprocessing, and output modified data. The two tabs used to edit tags and preprocess data are described here in the subsequent sections. Details for the Analyses and Synchrony tabs will be provided elsewhere, pending further development.

#### 2.2.1. Common Tab Controls

Several components and functions are common to both the Edit Tags and Preprocess Data tabs, such as the browse button, which can load one or more files. Drop-down lists at the top of each tab allow users to select which file and signal type to display. The timeseries graph and overlay are also common objects, so any edits made will carry over when transitioning between tabs.

#### 2.2.2. Edit Tags

The Edit Tags tab shown in Figure 5 is designed to allow users to add, delete, or annotate event tags in the raw data output by the LOTUS reader. Input signal data will be automatically plotted in the graph (blue line), with the tags marked by red vertical lines and numbered according to their sequence. A table is also provided, summarising the details for any event tags, including their order and the universal timestamp for the avro file containing the tag, as well as the date and the tag time to the nearest ms relative to the time zone selected in the LOTUS reader.

**Figure 6:**
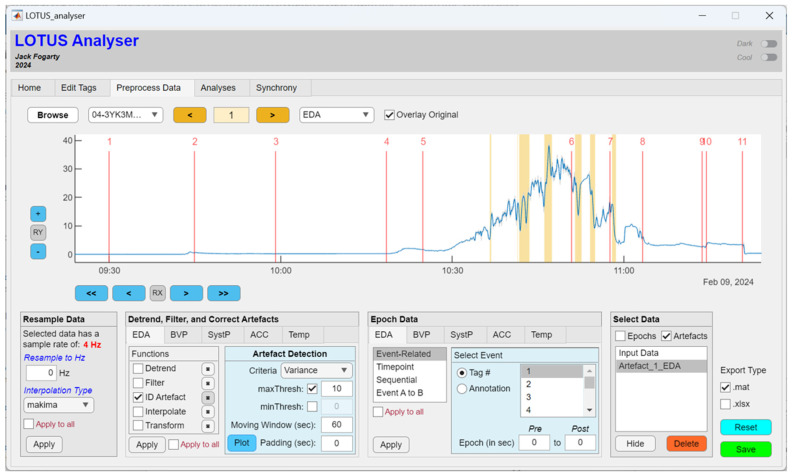
The LOTUS analyser, with the Preprocess Data tab showing detected EDA artefacts based on a maximum variance threshold (highlighted yellow).

Unique GUI controls in the Edit Tags tab allow the user to add new tags at a specific timepoint by setting the date, hour, minute, and ms and clicking add within the ‘Add Tags’ panel. An example is shown in Figure 5, where a new tag (Tag 11) has been added to the timeseries at 9:30 a.m. The details of new tags are added to the Event Tag table and stored in the data with an annotation recording when the new event was added. Tags can also be deleted in the ‘Remove Tags’ panel by selecting tags in the drop-down list and clicking delete. Changes to tags will first be highlighted in the plot and table—the user may then click ‘apply’ (bottom right) to finalise the changes; note that the apply button will also sort tags into their temporal order and update the tags’ labels accordingly (i.e., upon hitting apply, Tag 11 would become Tag 1 and the other tags’ labels would be adjusted, as shown in Figure 6). Clicking ‘reset’ will return the tags for the selected file back to their original state when first loaded into LOTUS analyser. The ‘save’ button will allow users to output modified data, with options to overwrite the tags in the current file.

#### 2.2.3. Preprocess Data

The Preprocess Data tab shown in Figure 6 was designed to provide a comprehensive suite of signal processing tools in one interface, with capacity for contributors to develop and build in extensions for these tools. The unique components of this tab include panel controls to (i) resample data; (ii) detrend, filter, and correct artefacts; (iii) epoch data; (iv) select data segments (e.g., epochs or artefacts); and (v) output modified data to a mat or Excel file (.xlsx).

##### Resample Data

The Resample Data panel allows users to specify a new sample rate for their signal data and the interpolation technique to be used in cases where up-sampling is required. Standard Matlab interpolation options are currently available to choose from, including linear, spline, pchip, and makima interpolations. The apply button will only resample the signal that is currently selected (i.e., shown in the plot and drop-down menu at the top of the GUI), unless the user ticks the ‘apply to all’ checkbox, in which case, the preprocessing will apply to all signals available for the current recording (e.g., EDA, BVP, and ACC), with the exception of systolic peaks.

##### Detrend, Filter, and Correct Artefact

The Detrend, Filter, and Correct Artefacts panel enables users to apply a variety of standard preprocessing techniques. Users can view and set different options and parameters by clicking the buttons beside each function. To apply the function, the checkbox for the selected process should be ticked before clicking apply. Detrending options include the inbuilt Matlab detrend function, as well as adapted functions to implement noise-tools detrend (nt_detrend) [40], and a dynamic detrending based on the smoothness priors typically used for HRV (sp_tarvainen) [41]. Filters are also available, including the moving average, moving median, and Butterworth filters commonly used with wearable signal data, with options to adjust parameters such as the window length, cut-offs, and filter order.

Artefact identification currently allows users to set thresholds based on the statistical properties of their signal data in terms of the amplitude, z-score, variance, standard deviation, and slope. Thresholding criteria can be computed in sliding windows and ‘padding’ can be added to reject a certain number of points around identified artefact. The plot button highlights any artefact in the timeseries graph (see Figure 6), whereas the apply button will directly remove those periods marked for rejection. Users may plot multiple types of artefacts, with each ‘plot’ of artefacts being added to the data selection panel list, enabling users to flexibly select, view, apply, or delete different artefact identification strategies with reference to the selected input data shown in the graph.

Interpolation options are included to fill gaps in the signals due to missing data or from artefact rejection. Several in-built Matlab functions are enabled for this, including the use of the previous, next, or nearest scores to fill gaps, as well as linear, spline, pchip, makima, moving average, or moving median functions. Finally, transform functions are made available, primarily to convert signal data to z-scores, computed across the entire recording or in a sliding window.

##### Epoch Data

The LOTUS analyser provides several options for epoching signal data, including (i) event-related, (ii) timepoint, (iii) sequential, and (iv) Event A to B epoching. Event-related and timepoint epoching allows users to segment data relative to a particular event tag or to a specific time (e.g., from 30 s before to 5 min after an event or time). Sequential epoching involves segmenting data into a series of epochs starting from a particular tag. Users may specify the number, length, overlap, and direction for sequential epochs to be generated (e.g., twenty 30 s epochs moving forward without overlap starting from Tag 2). Finally, A to B epoching can be used to isolate data between two tags. Users may navigate back to the Edit Tags tab to adjust or annotate tags to facilitate epoching as needed.

##### Exporting from the LOTUS Analyser

Data may be saved by selecting the required data in the Select Data panel and clicking save. Currently, users may choose to save the full preprocessed signal (i.e., ‘input data’) or epoched signals as a new mat file or an Excel file (.xlsx) for further processing and analysis.

## 3. Discussion

This article describes the first release of LOTUS, an open-source Matlab toolkit for longitudinal timeseries unification and signal processing. LOTUS addresses a critical gap in the software tools available for processing data from the Empatica EmbracePlus by offering a comprehensive toolkit integrating data reconstitution, preprocessing, and analysis within a user-friendly GUI. Existing tools, such as EmpaticaR and EmbracePlus-Visualiser, have limitations in processing the raw EmbracePlus avro data. In contrast, LOTUS simplifies wearable data management and enhances the analytical capabilities of EmbracePlus data through its GUI and flexible signal processing features.

As mentioned in Section 1, to date, most researchers have neglected to state their approach to processing EmbracePlus data. The lack of detail in previous studies could be due to the fact that researchers depend on the aggregate data provided by Empatica’s proprietary algorithms. In such a case, it would be difficult to provide explicit details regarding how the data are processed. However, it is possible that some researchers also utilise their own custom scripts to process raw data but leave this unstated. Indeed, this is evident in some existing reports, which indicate the use of R, Python, or Matlab to process data but refrain from going into detail.

Aggregate data from EmbracePlus can be easier to access and analyse than the raw data as the data are provided in CSV format and collapsed to a period of once per minute (i.e., a ~0.017 Hz sample rate). This can be valuable; however, utilising this form of output can also limit analyses and does not take advantage of the rich detail in raw EmbracePlus data. The processing of the aggregate data is also possible through proprietary algorithms and, therefore are difficult to report, replicate, or consider in terms of the methodological and scientific implications. Overall, there appears to be a need for improved standards of reporting in studies using wearable data from devices such as the EmbracePlus. It is hoped, then, that through LOTUS, researchers will be able to take advantage of raw EmbracePlus data more easily and thus reduce dependency on proprietary algorithms and strengthen the analysis and reporting of wearable physiological data; this will be critical as the field continues to grow.

Additional challenges and recommendations can be made in reviews of the existing research literature using the EmbracePlus. First and foremost, several studies indicate difficulties with data loss due to low-quality signals. Explanations for this are mostly attributed to movement or poor fitting of the device. This may be mitigated by having researchers or professionals check the fit of the wearable or training the participants in correct use. It should also be noted that Empatica algorithms will exclude periods from the aggregated data that are deemed to involve artefacts. The quality of this artefact rejection and whether data may be salvaged from the raw signal with other approaches is unclear. With LOTUS, researchers may inspect raw data, implement alternative preprocessing steps, and potentially find more conservative processes for their dataset to mitigate data loss.

Another challenge noted in previous studies relates to the classification of data. Studies using machine learning approaches often need to classify segments of recorded data to establish the ground truth (e.g., [4,5,8,13,16]). Annotation may be difficult without a tool to pinpoint (or add) event markers and annotations. Interpretation of the aggregate data may also be complicated without viewing or extracting precise periods from the underlying signal. LOTUS can assist with these processes by providing researchers with a tool to edit and annotate events, visualise, and segment data. This is also expected to assist with data synchronization across different modalities by allowing researchers to edit, resample, and segment data at a much higher temporal resolution.

Finally, it is notable that a wide range of wrist-based physiological markers are used to measure processes such as stress, fatigue, and emotion (e.g., EDA, PR, PRV). The Empatica shows promise for such research; however, there is little consistency in the extant literature regarding which wrist-based biomarkers to use, or how to interpret and consolidate inconsistent arousal patterns across the different physiological measures within and between studies. Interpretation of these measures is often context-specific, but with growing research using wearables to assess processes such as stress and emotional arousal, it will become more important to develop coherent and valid frameworks to guide the analysis, interpretation, and synthesis of the various wearable measures.

## 4. Conclusions

LOTUS provides the most comprehensive tool for EmbracePlus data processing to date. However, this toolkit is still considered a work in progress, with many features that may yet be developed or optimised. LOTUS would benefit from integrating a wider set of advanced modality-specific preprocessing functions. Moreover, while there is a focus on the EmbracePlus, it is also intended for this toolkit to be compatible with data from other devices. These additions can be built in over time to develop a more complete processing suite for wearable data. Moving forward, it is hoped that the recommendations in this article are useful, and that LOTUS can be refined through collaboration and feedback from users in the broader research community, while adding value to the burgeoning research using wearable sensors to study human health and behaviour.

## Figures and Tables

**Table 1:** Current functionality of known GitHub Toolkits compatible with EmbracePlus.

Toolbox Name	Software	Read	Reconstitute	Preprocessing
Wearables ^1,2,3^	R	X		
EmpaticaR ^4^	R	X		
EmbracePlus-Visualiser ^5^	Python	X	X	
LOTUS ^6^	Matlab	X	X	X
^1^ [https://github.com/PCdLf/wearables, accessed on 16 September 2024] ^2^ [https://github.com/PCdLf/wearalyze, accessed on 16 September 2024] ^3^ [https://github.com/PCdLf/wearables_international, accessed on 16 September 2024] ^4^ [https://github.com/brian-lau/empaticaR, accessed on 16 September 2024] ^5^ [https://github.com/JocelynVelarde/EmbracePlus-Visualizer, accessed on 16 September 2024] ^6^ [https://github.com/jack-fogarty/LOTUS, accessed on 6 October 2024]				

## Data Availability

LOTUS is publicly available for download at the following link: https://github.com/jack-fogarty/LOTUS (accessed on 6 October 2024).
